# Supplementary material for: Genomic Landscapes of Early-Onset Versus Average-Onset Colorectal Cancer Populations
Source: Cancers (Basel). 2025 Feb 28;17(5):836. doi: 10.3390/cancers17050836 (PMC11899610; doi:10.3390/cancers17050836)

Supplemental S1: Hereditary cancer genes evaluated as part of Tempus xT profiling.

“**Note**: germline findings are restricted to patients with T/N matched sequencing and the 46 genes consistent across incidental germline panels*: APC, ATM, AXIN2, BMPR1A, BRCA1, BRCA2, BRIP1, CDH1, CDKN2A, CEBPA, CHEK2, EGFR, EPCAM, ETV6, FH, FLCN, GATA2, MEN1, MLH1, MSH2, MSH3, MSH6, MUTYH, NBN, NF2, PALB2, PMS2, POLD1, POLE, PTEN, RAD51C, RAD51D, RB1, RET, RUNX1, SDHAF2, SDHB, SDHC, SDHD, SMAD4, STK11, TP53, TSC1, TSC2, VHL, WT1”

Supplemental S2: Immune cell infiltration among patients with early-onset (eoCRC) and average-onset (aoCRC) colorectal cancer who completed RNA sequencing.

	**Overall**, N = 9,218[a]	**eoCRC**, N = 1,997[a]	**aoCRC**, N = 7,221[a]	***p*-value**[b]
**% immune cells of all cells in the sample**	0.14 (0.09, 0.19)	0.14 (0.09, 0.19)	0.14 (0.10, 0.19)	0.029
Unknown	38	7	31	
**% B cells of all immune cells**	0.16 (0.07, 0.26)	0.16 (0.07, 0.25)	0.16 (0.07, 0.27)	0.042
Unknown	38	7	31	
**% CD4 T cells of all immune cells**	0.29 (0.21, 0.38)	0.31 (0.23, 0.40)	0.28 (0.20, 0.37)	<0.001
Unknown	38	7	31	
**% CD8 T cells of all immune cells**	0.03 (0.00, 0.08)	0.03 (0.00, 0.08)	0.03 (0.00, 0.08)	0.11
Unknown	38	7	31	
**% macrophages of all immune cells**	0.34 (0.23, 0.47)	0.33 (0.23, 0.46)	0.34 (0.23, 0.47)	0.09
Unknown	38	7	31	
**% NK cells of all immune cells**	0.11 (0.07, 0.16)	0.10 (0.07, 0.15)	0.11 (0.07, 0.16)	<0.001
Unknown	38	7	31	

[a]Median (interquartile range)

[b]Wilcox rank sum test

Supplemental S3: Figure depicting immune cell infiltration among patients with eoCRC (blue) and patients with aoCRC (yellow) who completed RNA sequencing.

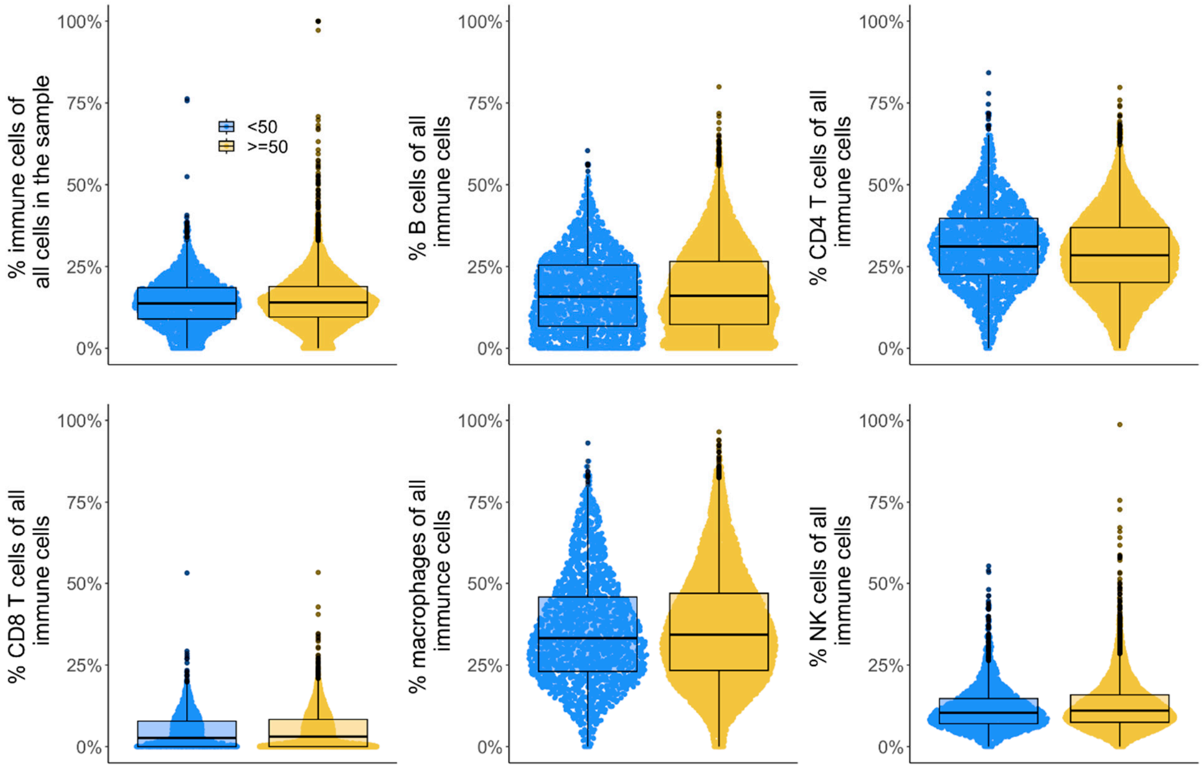

Supplemental S4: Oncoplot of 30 most common somatic alterations occurring in patients with eoCRC.

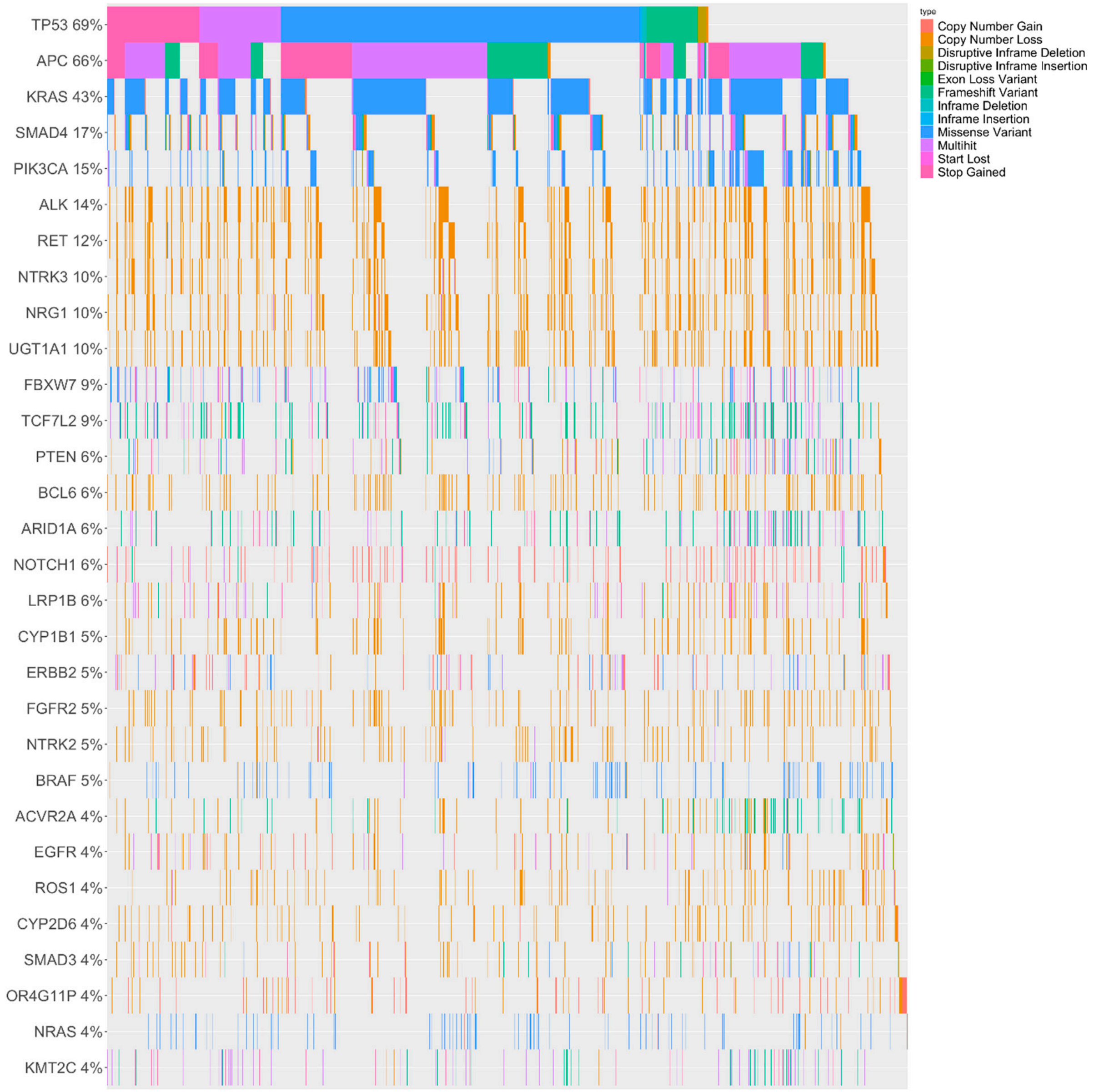

Supplemental S5: Oncoplot of 30 most common somatic alterations occurring in patients with aoCRC.

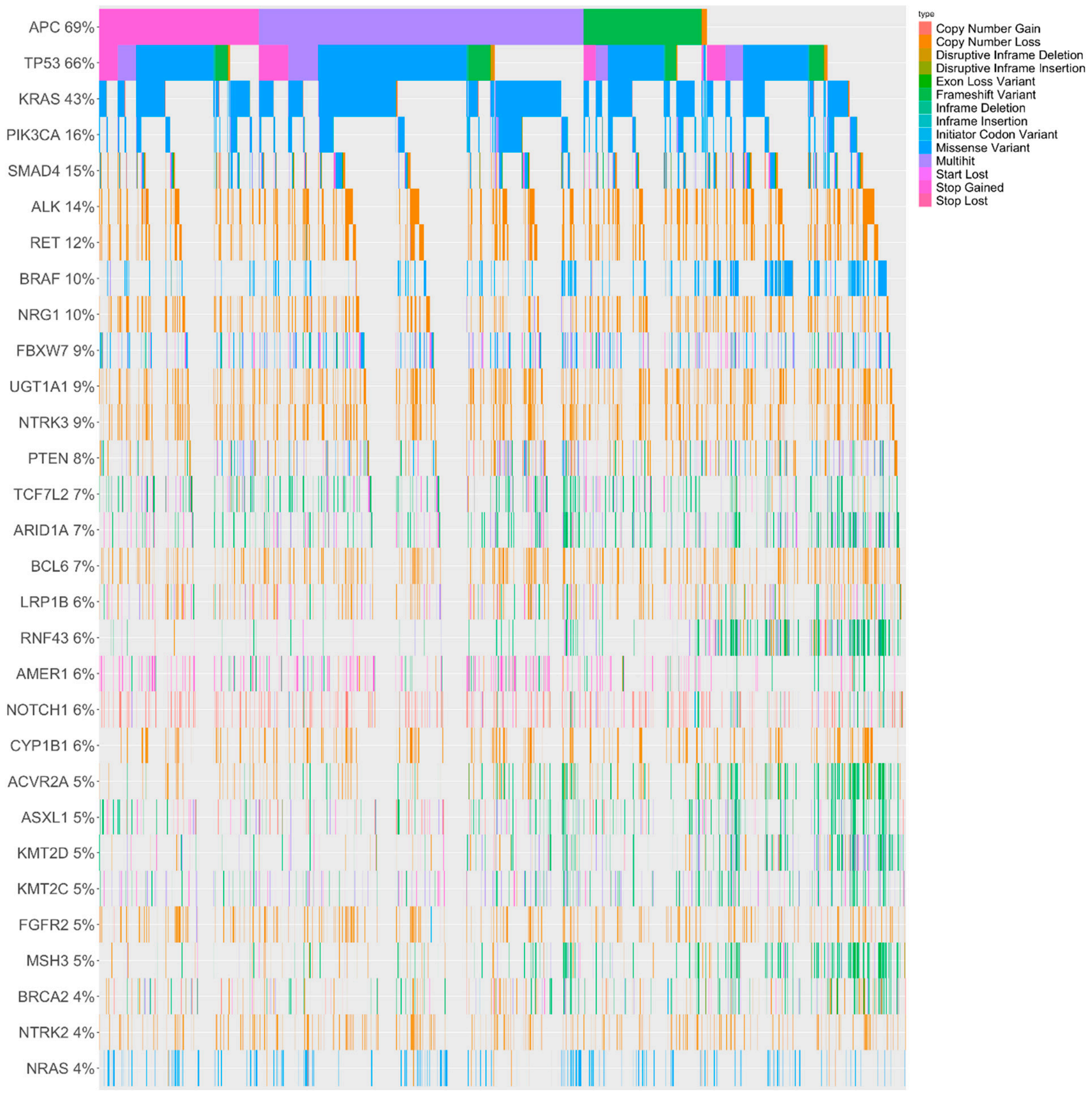

Supplemental S6: Comprehensive list of somatic alterations in patients with eoCRC and aoCRC by significance of q-value.

	**eoCRC,** N = 2,379	**aoCRC**, N = 8,627	***p*-value**[a]	**q-value**[b]
BRAF	111 (4.7%)	845 (9.8%)	<0.001	<0.001
RNF43	68 (2.9%)	515 (6.0%)	<0.001	<0.001
AMER1	83 (3.5%)	503 (5.8%)	<0.001	<0.001
ZNRF3	24 (1.0%)	192 (2.2%)	<0.001	0.008
UGT2B28	40 (1.7%)	71 (0.8%)	<0.001	0.009
RPL22	24 (1.0%)	180 (2.1%)	<0.001	0.02
NKX2-1	84 (3.5%)	199 (2.3%)	<0.001	0.022
C15orf40	63 (2.6%)	139 (1.6%)	<0.001	0.022
MSH6	54 (2.3%)	312 (3.6%)	0.001	0.024
GNAS	27 (1.1%)	187 (2.2%)	0.001	0.024
HNF1A	23 (1.0%)	168 (1.9%)	0.001	0.024
FLCN	50 (2.1%)	289 (3.3%)	0.002	0.027
SEC31A	39 (1.6%)	77 (0.9%)	0.002	0.027
FAS	14 (0.6%)	119 (1.4%)	0.002	0.027
ASXL1	83 (3.5%)	426 (4.9%)	0.003	0.036
CDK12	70 (2.9%)	167 (1.9%)	0.003	0.036
PRSS1	30 (1.3%)	56 (0.6%)	0.003	0.036
SMAD4	409 (17%)	1,274 (15%)	0.004	0.043
ERBB2	129 (5.4%)	350 (4.1%)	0.004	0.043
SMARCE1	24 (1.0%)	43 (0.5%)	0.005	0.049
MSH3	77 (3.2%)	391 (4.5%)	0.006	0.056
KEL	36 (1.5%)	78 (0.9%)	0.009	0.089
ACTA2	13 (0.5%)	99 (1.1%)	0.01	0.089
TP53	1,634 (69%)	5,688 (66%)	0.012	0.093
APC	1,567 (66%)	5,916 (69%)	0.012	0.093
POLE	59 (2.5%)	146 (1.7%)	0.012	0.093
FGF5	47 (2.0%)	111 (1.3%)	0.012	0.093
KDR	44 (1.8%)	102 (1.2%)	0.012	0.093
FLT4	80 (3.4%)	211 (2.4%)	0.014	0.1
SOX9	19 (0.8%)	125 (1.4%)	0.013	0.1
KMT2D	88 (3.7%)	422 (4.9%)	0.014	0.1
CHD2	23 (1.0%)	142 (1.6%)	0.016	0.1
PTEN	145 (6.1%)	648 (7.5%)	0.018	0.12
TCF7L2	203 (8.5%)	614 (7.1%)	0.02	0.12
OR4G11P	90 (3.8%)	247 (2.9%)	0.021	0.12
AASDH	35 (1.5%)	80 (0.9%)	0.021	0.12
CLOCK	35 (1.5%)	80 (0.9%)	0.021	0.12
TSHR	24 (1.0%)	50 (0.6%)	0.023	0.13
PML	80 (3.4%)	217 (2.5%)	0.024	0.13

EGFR	106 (4.5%)	300 (3.5%)	0.025	0.13
QKI	26 (1.1%)	56 (0.6%)	0.026	0.13
ZFHX3	26 (1.1%)	149 (1.7%)	0.029	0.14
NTRK3	239 (10%)	747 (8.7%)	0.036	0.2
IDH1	17 (0.7%)	105 (1.2%)	0.038	0.2
BCLAF1	24 (1.0%)	53 (0.6%)	0.041	0.2
KMT2C	90 (3.8%)	410 (4.8%)	0.044	0.2
ABRAXAS1	38 (1.6%)	94 (1.1%)	0.044	0.2
PTCH1	24 (1.0%)	133 (1.5%)	0.052	0.2
FGF6	58 (2.4%)	158 (1.8%)	0.059	0.3
HDAC2	36 (1.5%)	183 (2.1%)	0.06	0.3
APLNR	41 (1.7%)	106 (1.2%)	0.063	0.3
KLLN	28 (1.2%)	148 (1.7%)	0.064	0.3
ARID1B	38 (1.6%)	190 (2.2%)	0.067	0.3
FGF23	48 (2.0%)	128 (1.5%)	0.066	0.3
TOP2A	25 (1.1%)	59 (0.7%)	0.069	0.3
LATS1	27 (1.1%)	65 (0.8%)	0.07	0.3
JAK1	29 (1.2%)	150 (1.7%)	0.076	0.3
CDKN1B	68 (2.9%)	310 (3.6%)	0.081	0.3
CIC	32 (1.3%)	162 (1.9%)	0.08	0.3
MSH2	37 (1.6%)	96 (1.1%)	0.08	0.3
ARID1A	140 (5.9%)	592 (6.9%)	0.09	0.3
ACVR2A	107 (4.5%)	462 (5.4%)	0.094	0.3
BRCA2	86 (3.6%)	380 (4.4%)	0.09	0.3
ARID2	61 (2.6%)	279 (3.2%)	0.095	0.3
MCL1	34 (1.4%)	88 (1.0%)	0.092	0.3
GRID2	29 (1.2%)	73 (0.8%)	0.093	0.3
OR4F5	58 (2.4%)	165 (1.9%)	0.11	0.3
CREBBP	37 (1.6%)	179 (2.1%)	0.11	0.3
CASP8	33 (1.4%)	162 (1.9%)	0.11	0.3
RNLS	21 (0.9%)	111 (1.3%)	0.11	0.3
PIK3R1	66 (2.8%)	295 (3.4%)	0.12	0.3
PAX3	37 (1.6%)	100 (1.2%)	0.12	0.4
PDGFRA	61 (2.6%)	177 (2.1%)	0.13	0.4
PIK3CA	345 (15%)	1,358 (16%)	0.14	0.4
EPCAM	30 (1.3%)	146 (1.7%)	0.14	0.4
PHOX2B	51 (2.1%)	147 (1.7%)	0.2	0.4
HSPH1	38 (1.6%)	177 (2.1%)	0.2	0.4
GLI2	34 (1.4%)	93 (1.1%)	0.2	0.4
B2M	46 (1.9%)	209 (2.4%)	0.2	0.4
BCORL1	42 (1.8%)	193 (2.2%)	0.2	0.4
SMAD2	62 (2.6%)	272 (3.2%)	0.2	0.4
NOTCH2	61 (2.6%)	268 (3.1%)	0.2	0.4

SETBP1	19 (0.8%)	97 (1.1%)	0.2	0.4
DPYD	73 (3.1%)	222 (2.6%)	0.2	0.5
CYP2D6	96 (4.0%)	300 (3.5%)	0.2	0.5
MYB	68 (2.9%)	207 (2.4%)	0.2	0.5
KIT	52 (2.2%)	154 (1.8%)	0.2	0.5
EP300	29 (1.2%)	136 (1.6%)	0.2	0.5
HNMT	36 (1.5%)	102 (1.2%)	0.2	0.5
PTCH2	23 (1.0%)	112 (1.3%)	0.2	0.5
TET2	56 (2.4%)	168 (1.9%)	0.2	0.5
CKS1B	36 (1.5%)	103 (1.2%)	0.2	0.5
PTPN13	58 (2.4%)	176 (2.0%)	0.2	0.5
NCOR1	29 (1.2%)	134 (1.6%)	0.2	0.5
MS4A1	33 (1.4%)	94 (1.1%)	0.2	0.5
STC1	41 (1.7%)	121 (1.4%)	0.2	0.5
RB1	32 (1.3%)	145 (1.7%)	0.2	0.5
MLH1	37 (1.6%)	108 (1.3%)	0.3	0.5
WNK1	28 (1.2%)	79 (0.9%)	0.3	0.5
MYC	47 (2.0%)	141 (1.6%)	0.3	0.5
ATM	85 (3.6%)	352 (4.1%)	0.3	0.5
TCL1A	42 (1.8%)	125 (1.4%)	0.3	0.5
TTI2	41 (1.7%)	123 (1.4%)	0.3	0.6
FGF10	30 (1.3%)	87 (1.0%)	0.3	0.6
USF3	29 (1.2%)	84 (1.0%)	0.3	0.6
FGFR2	124 (5.2%)	406 (4.7%)	0.3	0.6
PRKN	41 (1.7%)	124 (1.4%)	0.3	0.6
GATA4	32 (1.3%)	141 (1.6%)	0.3	0.6
SETD2	31 (1.3%)	137 (1.6%)	0.3	0.6
RAD50	51 (2.1%)	215 (2.5%)	0.3	0.6
LRP1B	134 (5.6%)	531 (6.2%)	0.3	0.6
NTRK2	111 (4.7%)	364 (4.2%)	0.3	0.6
BCL6	145 (6.1%)	572 (6.6%)	0.3	0.7
BCOR	46 (1.9%)	194 (2.2%)	0.4	0.7
FBXW7	210 (8.8%)	814 (9.4%)	0.4	0.7
BCL11B	37 (1.6%)	113 (1.3%)	0.4	0.7
CDX2	34 (1.4%)	103 (1.2%)	0.4	0.7
ACVR1B	30 (1.3%)	90 (1.0%)	0.4	0.7
ROS1	99 (4.2%)	325 (3.8%)	0.4	0.7
ELF3	33 (1.4%)	142 (1.6%)	0.4	0.7
RARA	25 (1.1%)	74 (0.9%)	0.4	0.7
CTCF	33 (1.4%)	101 (1.2%)	0.4	0.7
EPHB1	34 (1.4%)	105 (1.2%)	0.4	0.7
NF1	58 (2.4%)	236 (2.7%)	0.4	0.7
H4C5	33 (1.4%)	102 (1.2%)	0.4	0.7

ADAM9	26 (1.1%)	79 (0.9%)	0.4	0.7
AKT1	26 (1.1%)	79 (0.9%)	0.4	0.7
PPP2R2A	36 (1.5%)	113 (1.3%)	0.4	0.7
MUTYH	28 (1.2%)	119 (1.4%)	0.4	0.7
ATR	27 (1.1%)	115 (1.3%)	0.4	0.7
PTPRT	47 (2.0%)	192 (2.2%)	0.5	0.7
CDKN2A	52 (2.2%)	168 (1.9%)	0.5	0.7
MGMT	37 (1.6%)	117 (1.4%)	0.5	0.7
MAP2K1	29 (1.2%)	90 (1.0%)	0.5	0.7
NRAS	90 (3.8%)	354 (4.1%)	0.5	0.7
CDKN2B	74 (3.1%)	294 (3.4%)	0.5	0.7
CCND2	45 (1.9%)	145 (1.7%)	0.5	0.7
SLIT2	40 (1.7%)	128 (1.5%)	0.5	0.7
BCR	27 (1.1%)	84 (1.0%)	0.5	0.7
POLR1D	25 (1.1%)	77 (0.9%)	0.5	0.7
MEF2C	28 (1.2%)	117 (1.4%)	0.5	0.7
FLT3	80 (3.4%)	267 (3.1%)	0.5	0.7
MTAP	28 (1.2%)	88 (1.0%)	0.5	0.7
BARD1	38 (1.6%)	155 (1.8%)	0.5	0.8
UGT1A1	234 (9.8%)	811 (9.4%)	0.5	0.8
FGFR1	74 (3.1%)	247 (2.9%)	0.5	0.8
DYNC2H1	41 (1.7%)	166 (1.9%)	0.5	0.8
ESR1	52 (2.2%)	171 (2.0%)	0.5	0.8
TP63	29 (1.2%)	93 (1.1%)	0.6	0.8
BRCA1	24 (1.0%)	76 (0.9%)	0.6	0.8
IDO1	24 (1.0%)	76 (0.9%)	0.6	0.8
INPP4B	28 (1.2%)	90 (1.0%)	0.6	0.8
HSPA1B	23 (1.0%)	95 (1.1%)	0.6	0.8
FGFR3	90 (3.8%)	307 (3.6%)	0.6	0.8
CTNNB1	59 (2.5%)	199 (2.3%)	0.6	0.8
MAP2K4	52 (2.2%)	204 (2.4%)	0.6	0.8
FLT1	51 (2.1%)	200 (2.3%)	0.6	0.8
MYL1	51 (2.1%)	200 (2.3%)	0.6	0.8
CDK8	40 (1.7%)	159 (1.8%)	0.6	0.8
CXCR4	36 (1.5%)	118 (1.4%)	0.6	0.8
RBM10	34 (1.4%)	112 (1.3%)	0.6	0.8
NOTCH3	30 (1.3%)	121 (1.4%)	0.6	0.8
KDM5A	32 (1.3%)	105 (1.2%)	0.6	0.8
TIGIT	28 (1.2%)	91 (1.1%)	0.6	0.8
HTRA4	25 (1.1%)	80 (0.9%)	0.6	0.8
ERCC3	53 (2.2%)	207 (2.4%)	0.6	0.8
NRG1	238 (10%)	835 (9.7%)	0.6	0.8
WRN	35 (1.5%)	116 (1.3%)	0.6	0.8

GFRA2	24 (1.0%)	78 (0.9%)	0.6	0.8
ERBB4	57 (2.4%)	221 (2.6%)	0.6	0.8
NOTCH1	138 (5.8%)	480 (5.6%)	0.7	0.8
SYNE1	78 (3.3%)	268 (3.1%)	0.7	0.8
TNFAIP6	43 (1.8%)	145 (1.7%)	0.7	0.8
FOXP1	38 (1.6%)	149 (1.7%)	0.7	0.8
FCGR3A	37 (1.6%)	124 (1.4%)	0.7	0.8
SMARCA4	27 (1.1%)	89 (1.0%)	0.7	0.8
H1-4	36 (1.5%)	122 (1.4%)	0.7	0.8
H3C2	36 (1.5%)	122 (1.4%)	0.7	0.8
APOB	26 (1.1%)	87 (1.0%)	0.7	0.8
FHIT	24 (1.0%)	80 (0.9%)	0.7	0.8
RET	291 (12%)	1,034 (12%)	0.7	0.9
FAT1	48 (2.0%)	183 (2.1%)	0.8	0.9
TUSC3	43 (1.8%)	148 (1.7%)	0.8	0.9
CASR	30 (1.3%)	116 (1.3%)	0.8	0.9
NTRK1	25 (1.1%)	97 (1.1%)	0.8	0.9
SPEN	24 (1.0%)	93 (1.1%)	0.8	0.9
PMS2	78 (3.3%)	275 (3.2%)	0.8	>0.9
CDH1	30 (1.3%)	104 (1.2%)	0.8	>0.9
KDM6A	28 (1.2%)	97 (1.1%)	0.8	>0.9
UGT1A9	34 (1.4%)	128 (1.5%)	0.8	>0.9
CYP1B1	130 (5.5%)	479 (5.6%)	0.9	>0.9
TAP1	36 (1.5%)	135 (1.6%)	0.9	>0.9
TERT	35 (1.5%)	123 (1.4%)	0.9	>0.9
MAP3K1	32 (1.3%)	120 (1.4%)	0.9	>0.9
MLH3	26 (1.1%)	98 (1.1%)	0.9	>0.9
MKI67	33 (1.4%)	116 (1.3%)	0.9	>0.9
RASA1	52 (2.2%)	193 (2.2%)	0.9	>0.9
SMAD3	93 (3.9%)	332 (3.8%)	0.9	>0.9
PBRM1	39 (1.6%)	145 (1.7%)	0.9	>0.9
NBN	36 (1.5%)	133 (1.5%)	>0.9	>0.9
FGF4	45 (1.9%)	161 (1.9%)	>0.9	>0.9
ERCC6	26 (1.1%)	96 (1.1%)	>0.9	>0.9
TAP2	39 (1.6%)	140 (1.6%)	>0.9	>0.9
ERBB3	37 (1.6%)	135 (1.6%)	>0.9	>0.9
SCART1	32 (1.3%)	115 (1.3%)	>0.9	>0.9
PAX8	31 (1.3%)	111 (1.3%)	>0.9	>0.9
BUB3	29 (1.2%)	106 (1.2%)	>0.9	>0.9
HOXA11	29 (1.2%)	106 (1.2%)	>0.9	>0.9
ING1	25 (1.1%)	90 (1.0%)	>0.9	>0.9
ALK	343 (14%)	1,245 (14%)	>0.9	>0.9
KRAS	1,022 (43%)	3,707 (43%)	>0.9	>0.9

[a] Pearson's Chi-squared test

[b] False discovery rate correction for multiple testing

Supplemental S7: *BRAF* alterations in patients witheoCRC and aoCRC.

	**Overall**, N = 11,006	**eoCRC**, N = 2,379	**aoCRC**, N = 8,627	*p*-value[a]
**p.Val600Glu**	735 (6.7%)	71 (3.0%)	664 (7.7%)	<0.001
**p.Leu597Arg**	4 (<0.1%)	2 (<0.1%)	2 (<0.1%)	0.2
**p.Gly464Arg**	1 (<0.1%)	1 (<0.1%)	0 (0%)	0.2
**p.Asp594Ala**	1 (<0.1%)	1 (<0.1%)	0 (0%)	0.2
**p.Gly466Glu**	9 (<0.1%)	0 (0%)	9 (0.1%)	0.2
**p.Asn581Ile**	5 (<0.1%)	2 (<0.1%)	3 (<0.1%)	0.3
**p.Gly469Ala**	14 (0.1%)	1 (<0.1%)	13 (0.2%)	0.3
**p.Gly469Arg**	6 (<0.1%)	0 (0%)	6 (<0.1%)	0.4
**p.Asp594Gly**	54 (0.5%)	9 (0.4%)	45 (0.5%)	0.4
**p.Gly469Glu**	2 (<0.1%)	1 (<0.1%)	1 (<0.1%)	0.4
**p.Gly596Arg**	2 (<0.1%)	1 (<0.1%)	1 (<0.1%)	0.4
**p.Leu597Gln**	2 (<0.1%)	1 (<0.1%)	1 (<0.1%)	0.4
**p.Asp594Asn**	18 (0.2%)	2 (<0.1%)	16 (0.2%)	0.4
**p.Gly466Val**	11 (<0.1%)	1 (<0.1%)	10 (0.1%)	0.5
**p.Lys483Glu**	4 (<0.1%)	0 (0%)	4 (<0.1%)	0.6
**p.Gly466Arg**	5 (<0.1%)	0 (0%)	5 (<0.1%)	0.6
**p.Phe247Leu**	1 (<0.1%)	0 (0%)	1 (<0.1%)	>0.9
**p.Asp594Glu**	1 (<0.1%)	0 (0%)	1 (<0.1%)	>0.9
**p.Ser602Tyr**	1 (<0.1%)	0 (0%)	1 (<0.1%)	>0.9
**p.Glu501Lys**	1 (<0.1%)	0 (0%)	1 (<0.1%)	>0.9
**p.Arg603Gly**	1 (<0.1%)	0 (0%)	1 (<0.1%)	>0.9
**p.Asp594Val**	1 (<0.1%)	0 (0%)	1 (<0.1%)	>0.9
**p.Asn581His**	2 (<0.1%)	0 (0%)	2 (<0.1%)	>0.9
**p.Asn581Tyr**	1 (<0.1%)	0 (0%)	1 (<0.1%)	>0.9
**p.Asn581Thr**	1 (<0.1%)	0 (0%)	1 (<0.1%)	>0.9
**p.Gln257Arg**	1 (<0.1%)	0 (0%)	1 (<0.1%)	>0.9
**p.Gly593Cys**	1 (<0.1%)	0 (0%)	1 (<0.1%)	>0.9
**p.Arg558Gln**	1 (<0.1%)	0 (0%)	1 (<0.1%)	>0.9
**p.Gly596Asp**	1 (<0.1%)	0 (0%)	1 (<0.1%)	>0.9
**p.Lys483Gln**	1 (<0.1%)	0 (0%)	1 (<0.1%)	>0.9
**p.Gly464Glu**	1 (<0.1%)	0 (0%)	1 (<0.1%)	>0.9
**p.Thr599dup**	2 (<0.1%)	0 (0%)	2 (<0.1%)	>0.9
**p.Asn486_Ala489delinsArg**	1 (<0.1%)	0 (0%)	1 (<0.1%)	>0.9
**p.Arg506_Lys507insValLeuArg**	1 (<0.1%)	0 (0%)	1 (<0.1%)	>0.9
**p.ValLys600GluLys**	1 (<0.1%)	0 (0%)	1 (<0.1%)	>0.9
**p.LysSer601AsnSer**	1 (<0.1%)	0 (0%)	1 (<0.1%)	>0.9
**p.Val600Arg**	1 (<0.1%)	0 (0%)	1 (<0.1%)	>0.9
**p.Val600_Lys601delinsGlu**	1 (<0.1%)	0 (0%)	1 (<0.1%)	>0.9
**p.Gly469Val**	9 (<0.1%)	2 (<0.1%)	7 (<0.1%)	>0.9
**p.Gly464Val**	4 (<0.1%)	1 (<0.1%)	3 (<0.1%)	>0.9
**p.Phe595Leu**	3 (<0.1%)	0 (0%)	3 (<0.1%)	>0.9

**p.Val471Phe**	3 (<0.1%)	0 (0%)	3 (<0.1%)	>0.9
**p.Lys601Glu**	4 (<0.1%)	1 (<0.1%)	3 (<0.1%)	>0.9
**p.Asn581Ser**	6 (<0.1%)	1 (<0.1%)	5 (<0.1%)	>0.9
**p.Gly466Ala**	3 (<0.1%)	0 (0%)	3 (<0.1%)	>0.9

[a] Pearson's Chi-squared test; Fisher's exact test

Supplemental S8: Comprehensive list of *KRAS* alterations in patients with eoCRC and aoCRC.

	**Overall**, N = 11,006	**eoCRC**, N = 2,379	**aoCRC**, N = 8,627	*p*-value[a]
**p.GlyGln60GlyLys**	35 (0.3%)	0 (0%)	35 (0.4%)	0.002
**p.Ala146Val**	56 (0.5%)	6 (0.3%)	50 (0.6%)	0.047
**p.Lys117Asn**	53 (0.5%)	16 (0.7%)	37 (0.4%)	0.13
**p.Ala59Thr**	14 (0.1%)	5 (0.2%)	9 (0.1%)	0.2
**p.Ala146Gly**	1 (<0.1%)	1 (<0.1%)	0 (0%)	0.2
**p.Lys147Gln**	1 (<0.1%)	1 (<0.1%)	0 (0%)	0.2
**p.Pro34Leu**	1 (<0.1%)	1 (<0.1%)	0 (0%)	0.2
**p.GlnGlu61LeuGln**	1 (<0.1%)	1 (<0.1%)	0 (0%)	0.2
**p.Gly12Ser**	184 (1.7%)	33 (1.4%)	151 (1.8%)	0.2
**p.Gly12Asp**	1,401 (13%)	320 (13%)	1,081 (13%)	0.2
**p.Ala146Pro**	10 (<0.1%)	4 (0.2%)	6 (<0.1%)	0.2
**p.Gln61Arg**	24 (0.2%)	3 (0.1%)	21 (0.2%)	0.3
**p.Gly13Val**	5 (<0.1%)	2 (<0.1%)	3 (<0.1%)	0.3
**p.Ala11_Gly12dup**	2 (<0.1%)	1 (<0.1%)	1 (<0.1%)	0.4
**p.Gln61His**	134 (1.2%)	25 (1.1%)	109 (1.3%)	0.4
**p.Ala59Gly**	3 (<0.1%)	1 (<0.1%)	2 (<0.1%)	0.5
**p.Gly13Cys**	40 (0.4%)	7 (0.3%)	33 (0.4%)	0.5
**p.Gly12Val**	998 (9.1%)	208 (8.7%)	790 (9.2%)	0.5
**p.Gln61Leu**	39 (0.4%)	10 (0.4%)	29 (0.3%)	0.5
**p.Val14Ile**	15 (0.1%)	4 (0.2%)	11 (0.1%)	0.5
**p.Asp33Glu**	4 (<0.1%)	0 (0%)	4 (<0.1%)	0.6
**p.Gly12Cys**	324 (2.9%)	74 (3.1%)	250 (2.9%)	0.6
**p.Gly12Phe**	6 (<0.1%)	2 (<0.1%)	4 (<0.1%)	0.6
**p.Ala146Thr**	248 (2.3%)	56 (2.4%)	192 (2.2%)	0.7
**p.Gln22Lys**	13 (0.1%)	2 (<0.1%)	11 (0.1%)	0.7
**p.Gly12Ala**	231 (2.1%)	48 (2.0%)	183 (2.1%)	0.8
**p.Gly13Asp**	752 (6.8%)	164 (6.9%)	588 (6.8%)	0.9
**p.Gly12Arg**	42 (0.4%)	9 (0.4%)	33 (0.4%)	>0.9
**p.Gly60Asp**	2 (<0.1%)	0 (0%)	2 (<0.1%)	>0.9
**p.Arg68Ser**	2 (<0.1%)	0 (0%)	2 (<0.1%)	>0.9
**p.Asp57Asn**	2 (<0.1%)	0 (0%)	2 (<0.1%)	>0.9
**p.Gln61Pro**	1 (<0.1%)	0 (0%)	1 (<0.1%)	>0.9
**p.Lys147Asn**	1 (<0.1%)	0 (0%)	1 (<0.1%)	>0.9
**p.Asp119His**	1 (<0.1%)	0 (0%)	1 (<0.1%)	>0.9
**p.Lys147Glu**	1 (<0.1%)	0 (0%)	1 (<0.1%)	>0.9
**p.Glu63Lys**	1 (<0.1%)	0 (0%)	1 (<0.1%)	>0.9
**p.Thr58Ile**	1 (<0.1%)	0 (0%)	1 (<0.1%)	>0.9
**p.Ile36Met**	1 (<0.1%)	0 (0%)	1 (<0.1%)	>0.9
**p.Gln61Glu**	1 (<0.1%)	0 (0%)	1 (<0.1%)	>0.9
**p.Lys117Arg**	1 (<0.1%)	0 (0%)	1 (<0.1%)	>0.9

**p.Gly60Ser**	1 (<0.1%)	0 (0%)	1 (<0.1%)	>0.9
**p.GlyGly12GlyArg**	1 (<0.1%)	0 (0%)	1 (<0.1%)	>0.9
**p.Gly12Ile**	1 (<0.1%)	0 (0%)	1 (<0.1%)	>0.9
**p.GlyGly12AspCys**	1 (<0.1%)	0 (0%)	1 (<0.1%)	>0.9
**p.Lys117Tyr**	1 (<0.1%)	0 (0%)	1 (<0.1%)	>0.9
**p.AlaGly11ProAsp**	1 (<0.1%)	0 (0%)	1 (<0.1%)	>0.9
**p.Gly12_Gly13dup**	1 (<0.1%)	0 (0%)	1 (<0.1%)	>0.9
**p.Gly12Leu**	1 (<0.1%)	0 (0%)	1 (<0.1%)	>0.9
**p.Gly12_Gly13insAlaGly**	2 (<0.1%)	0 (0%)	2 (<0.1%)	>0.9
**p.GlnGlu61HisAsp**	1 (<0.1%)	0 (0%)	1 (<0.1%)	>0.9
**p.GlyGly12AlaArg**	1 (<0.1%)	0 (0%)	1 (<0.1%)	>0.9
**p.Gly60Val**	3 (<0.1%)	0 (0%)	3 (<0.1%)	>0.9
**p.Leu19Phe**	10 (<0.1%)	2 (<0.1%)	8 (<0.1%)	>0.9
**p.Gly13Arg**	9 (<0.1%)	2 (<0.1%)	7 (<0.1%)	>0.9
**p.Phe156Leu**	3 (<0.1%)	0 (0%)	3 (<0.1%)	>0.9
**p.Ala59Glu**	4 (<0.1%)	1 (<0.1%)	3 (<0.1%)	>0.9
**p.Gly13dup**	4 (<0.1%)	1 (<0.1%)	3 (<0.1%)	>0.9
**p.GlyVal13AspIle**	3 (<0.1%)	0 (0%)	3 (<0.1%)	>0.9
**p.Gly10dup**	3 (<0.1%)	0 (0%)	3 (<0.1%)	>0.9
**p.Gly12Trp**	3 (<0.1%)	0 (0%)	3 (<0.1%)	>0.9
**p.Gly13Glu**	3 (<0.1%)	0 (0%)	3 (<0.1%)	>0.9

[a] Pearson's Chi-squared test

Supplemental S9: Frequency of copy number variants (including amplifications and deletions) among patients with eoCRC and aoCRC

	**eoCRC,** N=2,379	**aoCRC,** N=8,627	**p-value**[a]	**q-value**[b]
**ERBB2**	88 (3.7%)	198 (2.3%)	<0.001	0.015
**NKX2-1**	84 (3.5%)	198 (2.3%)	<0.001	0.037
**CDK12**	54 (2.3%)	115 (1.3%)	0.001	0.037
**PRSS1**	30 (1.3%)	56 (0.6%)	0.003	0.075
**SMARCE1**	24 (1.0%)	43 (0.5%)	0.005	0.1
**PTEN**	47 (2.0%)	262 (3.0%)	0.006	0.1
**EGFR**	100 (4.2%)	276 (3.2%)	0.017	0.14
**FLT4**	79 (3.3%)	211 (2.4%)	0.018	0.14
**FGF5**	47 (2.0%)	111 (1.3%)	0.012	0.14
**KDR**	43 (1.8%)	99 (1.1%)	0.012	0.14
**BRCA2**	25 (1.1%)	152 (1.8%)	0.015	0.14
**ABRAXAS1**	37 (1.6%)	84 (1.0%)	0.016	0.14
**KEL**	35 (1.5%)	77 (0.9%)	0.013	0.14
**TET2**	35 (1.5%)	79 (0.9%)	0.018	0.14
**ACTA2**	13 (0.5%)	99 (1.1%)	0.01	0.14
**PML**	80 (3.4%)	217 (2.5%)	0.024	0.2
**QKI**	26 (1.1%)	56 (0.6%)	0.026	0.2
**CDKN2A**	39 (1.6%)	94 (1.1%)	0.03	0.2
**NTRK3**	238 (10%)	746 (8.6%)	0.04	0.2
**MS4A1**	31 (1.3%)	73 (0.8%)	0.041	0.2
**BCLAF1**	24 (1.0%)	53 (0.6%)	0.041	0.2
**APLNR**	41 (1.7%)	103 (1.2%)	0.044	0.2
**FGF6**	58 (2.4%)	158 (1.8%)	0.059	0.3
**FGF23**	48 (2.0%)	128 (1.5%)	0.066	0.3
**KLLN**	28 (1.2%)	148 (1.7%)	0.064	0.3
**TOP2A**	25 (1.1%)	59 (0.7%)	0.069	0.3
**PTPRT**	20 (0.8%)	111 (1.3%)	0.076	0.3
**MCL1**	34 (1.4%)	88 (1.0%)	0.092	0.4
**SETBP1**	17 (0.7%)	95 (1.1%)	0.1	0.4
**NOTCH2**	51 (2.1%)	237 (2.7%)	0.1	0.4
**PTPN13**	35 (1.5%)	93 (1.1%)	0.11	0.4
**PAX3**	37 (1.6%)	100 (1.2%)	0.12	0.4
**PDGFRA**	59 (2.5%)	172 (2.0%)	0.14	0.4
**PHOX2B**	51 (2.1%)	147 (1.7%)	0.2	0.4
**HSPH1**	38 (1.6%)	177 (2.1%)	0.2	0.4
**EPCAM**	30 (1.3%)	146 (1.7%)	0.14	0.4
**GLI2**	34 (1.4%)	93 (1.1%)	0.2	0.4

**BCL11B**	33 (1.4%)	90 (1.0%)	0.2	0.4
**PTCH2**	22 (0.9%)	112 (1.3%)	0.14	0.4
**NOTCH1**	126 (5.3%)	400 (4.6%)	0.2	0.5
**DPYD**	73 (3.1%)	222 (2.6%)	0.2	0.5
**CYP2D6**	96 (4.0%)	300 (3.5%)	0.2	0.5
**MYB**	68 (2.9%)	207 (2.4%)	0.2	0.5
**CKS1B**	36 (1.5%)	103 (1.2%)	0.2	0.5
**TCL1A**	42 (1.8%)	124 (1.4%)	0.2	0.6
**WNK1**	28 (1.2%)	79 (0.9%)	0.3	0.6
**KRAS**	27 (1.1%)	76 (0.9%)	0.3	0.6
**FGF10**	30 (1.3%)	86 (1.0%)	0.3	0.6
**MYC**	46 (1.9%)	139 (1.6%)	0.3	0.6
**FGFR2**	123 (5.2%)	401 (4.6%)	0.3	0.6
**KIT**	49 (2.1%)	150 (1.7%)	0.3	0.6
**HDAC2**	27 (1.1%)	122 (1.4%)	0.3	0.6
**SMAD4**	104 (4.4%)	338 (3.9%)	0.3	0.6
**GATA4**	32 (1.3%)	141 (1.6%)	0.3	0.6
**PRKN**	33 (1.4%)	98 (1.1%)	0.3	0.6
**BCL6**	145 (6.1%)	572 (6.6%)	0.3	0.7
**POLE**	40 (1.7%)	123 (1.4%)	0.4	0.7
**MAP2K4**	22 (0.9%)	99 (1.1%)	0.4	0.7
**NTRK2**	110 (4.6%)	363 (4.2%)	0.4	0.7
**RARA**	25 (1.1%)	74 (0.9%)	0.4	0.7
**EPHB1**	34 (1.4%)	104 (1.2%)	0.4	0.7
**ROS1**	98 (4.1%)	324 (3.8%)	0.4	0.7
**FLT3**	79 (3.3%)	260 (3.0%)	0.4	0.7
**SMAD3**	54 (2.3%)	174 (2.0%)	0.4	0.7
**DYNC2H1**	40 (1.7%)	166 (1.9%)	0.4	0.7
**MTAP**	26 (1.1%)	79 (0.9%)	0.4	0.7
**CDKN2B**	74 (3.1%)	294 (3.4%)	0.5	0.8
**MGMT**	37 (1.6%)	117 (1.4%)	0.5	0.8
**SLIT2**	25 (1.1%)	77 (0.9%)	0.5	0.8
**CCND2**	45 (1.9%)	145 (1.7%)	0.5	0.8
**UGT1A1**	234 (9.8%)	811 (9.4%)	0.5	0.8
**FGFR1**	74 (3.1%)	247 (2.9%)	0.5	0.8
**CDKN1B**	59 (2.5%)	235 (2.7%)	0.5	0.8
**BARD1**	29 (1.2%)	120 (1.4%)	0.5	0.8
**ARID2**	26 (1.1%)	109 (1.3%)	0.5	0.8
**FGFR3**	90 (3.8%)	306 (3.5%)	0.6	0.8
**LRP1B**	78 (3.3%)	305 (3.5%)	0.5	0.8
**ERBB4**	56 (2.4%)	220 (2.6%)	0.6	0.8

**FLT1**	51 (2.1%)	200 (2.3%)	0.6	0.8
**ESR1**	51 (2.1%)	168 (1.9%)	0.5	0.8
**ERCC3**	46 (1.9%)	183 (2.1%)	0.6	0.8
**CDK8**	40 (1.7%)	159 (1.8%)	0.6	0.8
**KDM5A**	32 (1.3%)	105 (1.2%)	0.6	0.8
**PPP2R2A**	30 (1.3%)	98 (1.1%)	0.6	0.8
**TP63**	29 (1.2%)	93 (1.1%)	0.6	0.8
**TIGIT**	28 (1.2%)	91 (1.1%)	0.6	0.8
**BCR**	26 (1.1%)	84 (1.0%)	0.6	0.8
**IDO1**	24 (1.0%)	76 (0.9%)	0.6	0.8
**WRN**	35 (1.5%)	116 (1.3%)	0.6	0.8
**NTRK1**	24 (1.0%)	97 (1.1%)	0.6	0.8
**SYNE1**	78 (3.3%)	267 (3.1%)	0.6	0.8
**FCGR3A**	37 (1.6%)	124 (1.4%)	0.7	0.8
**RASA1**	24 (1.0%)	96 (1.1%)	0.7	0.8
**NRG1**	237 (10.0%)	835 (9.7%)	0.7	0.8
**CXCR4**	34 (1.4%)	114 (1.3%)	0.7	0.8
**APC**	51 (2.1%)	175 (2.0%)	0.7	0.8
**RET**	288 (12%)	1,026 (12%)	0.8	0.9
**TUSC3**	43 (1.8%)	148 (1.7%)	0.8	0.9
**APOB**	25 (1.1%)	85 (1.0%)	0.8	0.9
**CASR**	30 (1.3%)	115 (1.3%)	0.8	0.9
**CYP1B1**	129 (5.4%)	476 (5.5%)	0.9	>0.9
**TAP1**	36 (1.5%)	135 (1.6%)	0.9	>0.9
**UGT1A9**	34 (1.4%)	128 (1.5%)	0.8	>0.9
**MKI67**	33 (1.4%)	116 (1.3%)	0.9	>0.9
**ALK**	342 (14%)	1,242 (14%)	>0.9	>0.9
**PMS2**	59 (2.5%)	215 (2.5%)	>0.9	>0.9
**FGF4**	45 (1.9%)	161 (1.9%)	>0.9	>0.9
**TAP2**	39 (1.6%)	140 (1.6%)	>0.9	>0.9
**PAX8**	31 (1.3%)	111 (1.3%)	>0.9	>0.9
**HOXA11**	29 (1.2%)	106 (1.2%)	>0.9	>0.9
**ERCC6**	26 (1.1%)	96 (1.1%)	>0.9	>0.9

[a] Pearson's Chi-squared test

[b] False discovery rate correction for multiple testing

Supplemental S10: Most frequent pathogenic germline variants in patients with eoCRC.

	**eoCRC** N = 1,413
MUTYH	18 (1.3%)
ATM	11 (0.8%)
APC	9 (0.6%)
CHEK2	7 (0.5%)
BRCA2	6 (0.4%)
MSH2	5 (0.4%)
TP53	5 (0.4%)
BRIP1	4 (0.3%)
MLH1	4 (0.3%)
MSH3	4 (0.3%)
RAD51C	4 (0.3%)
NBN	3 (0.2%)
PALB2	3 (0.2%)
PMS2	3 (0.2%)
RAD51D	3 (0.2%)
BRCA1	2 (0.1%)
FH	2 (0.1%)
MSH6	2 (0.1%)
RET	2 (0.1%)
VHL	2 (0.1%)
ETV6	1 (<0.1%)
FLCN	1 (<0.1%)

Supplemental S11: Most frequent pathogenic germline variants in patients with aoCRC.

	**aoCRC,** N = 4,898
MUTYH	84 (1.7%)
CHEK2	21 (0.4%)
ATM	19 (0.4%)
BRCA2	14 (0.3%)
MSH6	13 (0.3%)
MLH1	12 (0.2%)
PMS2	12 (0.2%)
APC	11 (0.2%)
BRCA1	9 (0.2%)
MSH2	8 (0.2%)
NBN	7 (0.1%)
PALB2	7 (0.1%)
MSH3	6 (0.1%)
BRIP1	5 (0.1%)
RAD51D	4 (<0.1%)
RAD51C	3 (<0.1%)
TSC2	3 (<0.1%)
VHL	3 (<0.1%)
CDH1	2 (<0.1%)
FH	2 (<0.1%)
FLCN	2 (<0.1%)
RET	2 (<0.1%)
TP53	2 (<0.1%)
CDKN2A	1 (<0.1%)
PTEN	1 (<0.1%)
SDHB	1 (<0.1%)
SMAD4	1 (<0.1%)

Supplemental S12: Pathogenic germline variants in patients with eoCRC and aoCRC by q-value.

	**eoCRC,** N = 1,413	**aoCRC,** N = 4,898	*p*-**value**[a]	*q*-**value**[b]
TP53	5 (0.4%)	2 (<0.1%)	0.008	0.2
APC	9 (0.6%)	11 (0.2%)	0.027	0.4
ATM	11 (0.8%)	19 (0.4%)	0.06	0.4
RAD51C	4 (0.3%)	3 (<0.1%)	0.049	0.4
MUTYH	18 (1.3%)	84 (1.7%)	0.2	0.6
MSH2	5 (0.4%)	8 (0.2%)	0.2	0.6
BRIP1	4 (0.3%)	5 (0.1%)	0.12	0.6
MSH3	4 (0.3%)	6 (0.1%)	0.2	0.6
RAD51D	3 (0.2%)	4 (<0.1%)	0.2	0.6
FH	2 (0.1%)	2 (<0.1%)	0.2	0.6
RET	2 (0.1%)	2 (<0.1%)	0.2	0.6
ETV6	1 (<0.1%)	0 (0%)	0.2	0.6
VHL	2 (0.1%)	3 (<0.1%)	0.3	0.7
BRCA2	6 (0.4%)	14 (0.3%)	0.4	0.8
NBN	3 (0.2%)	7 (0.1%)	0.5	0.8
PALB2	3 (0.2%)	7 (0.1%)	0.5	0.8
MSH6	2 (0.1%)	13 (0.3%)	0.5	0.8
FLCN	1 (<0.1%)	2 (<0.1%)	0.5	0.8
CHEK2	7 (0.5%)	21 (0.4%)	0.7	>0.9
MLH1	4 (0.3%)	12 (0.2%)	0.8	>0.9
PMS2	3 (0.2%)	12 (0.2%)	>0.9	>0.9
BRCA1	2 (0.1%)	9 (0.2%)	>0.9	>0.9
TSC2	0 (0%)	3 (<0.1%)	>0.9	>0.9
CDH1	0 (0%)	2 (<0.1%)	>0.9	>0.9
CDKN2A	0 (0%)	1 (<0.1%)	>0.9	>0.9
PTEN	0 (0%)	1 (<0.1%)	>0.9	>0.9
SDHB	0 (0%)	1 (<0.1%)	>0.9	>0.9
SMAD4	0 (0%)	1 (<0.1%)	>0.9	>0.9

[a] Pearson's Chi-squared test

[b] False discovery rate correction for multiple testing

Supplemental S13: High penetrance pathogenic germline variants in patients with eoCRC and aoCRC by q-value.

	**eoCRC,** N = 1,413[1]	**aoCRC,** N = 4,898[1]	***p*-value**[a]	***q*-value**[b]
TP53	5 (0.4%)	2 (<0.1%)	0.008	0.2
APC	9 (0.6%)	11 (0.2%)	0.027	0.3
MUTYH	18 (1.3%)	84 (1.7%)	0.2	>0.9
MSH2	5 (0.4%)	8 (0.2%)	0.2	>0.9
FH	2 (0.1%)	2 (<0.1%)	0.2	>0.9
RET	2 (0.1%)	2 (<0.1%)	0.2	>0.9
VHL	2 (0.1%)	3 (<0.1%)	0.3	>0.9
BRCA2	6 (0.4%)	14 (0.3%)	0.4	>0.9
MLH1	4 (0.3%)	12 (0.2%)	0.8	>0.9
MSH6	2 (0.1%)	13 (0.3%)	0.5	>0.9
PMS2	3 (0.2%)	12 (0.2%)	>0.9	>0.9
PALB2	3 (0.2%)	7 (0.1%)	0.5	>0.9
BRCA1	2 (0.1%)	9 (0.2%)	>0.9	>0.9
FLCN	1 (<0.1%)	2 (<0.1%)	0.5	>0.9
SDHA	1 (<0.1%)	3 (<0.1%)	>0.9	>0.9
PTCH1	0 (0%)	3 (<0.1%)	>0.9	>0.9
TSC2	0 (0%)	3 (<0.1%)	>0.9	>0.9
CDH1	0 (0%)	2 (<0.1%)	>0.9	>0.9
CDKN2A	0 (0%)	1 (<0.1%)	>0.9	>0.9
PTEN	0 (0%)	1 (<0.1%)	>0.9	>0.9
SDHB	0 (0%)	1 (<0.1%)	>0.9	>0.9
SMAD4	0 (0%)	1 (<0.1%)	>0.9	>0.9

[a] Pearson's Chi-squared test

[b] False discovery rate correction for multiple testing

Supplemental S14: Pathogenic germline variants in eoCRC and aoCRC patients with **a.** MSI-L/MSS tumors and **b.** MSI-H/dMMR tumors.

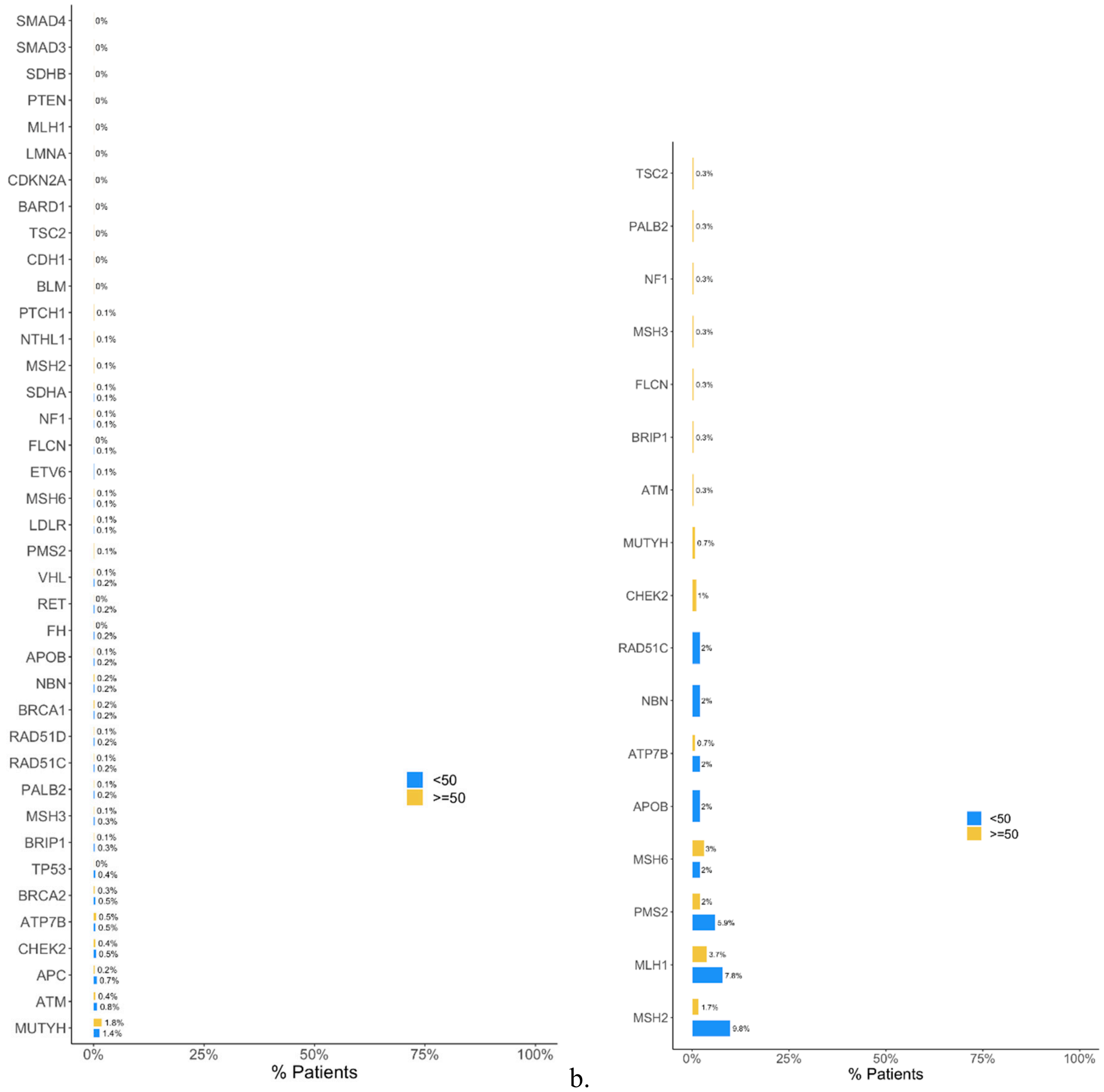

a. b.

Supplemental S15: Somatic mutation profile of patients with eoCRC and aoCRC who were incidentally found to have a pathogenic or likely pathogenic germline variant in a Lynch syndrome gene.

	**eoCRC**, N = 14	**aoCRC,** N = 45	***p*-value**[a]	***q*-value**[b]
APC	10 (71%)	32 (71%)	>0.9	>0.9
ACVR2A	9 (64%)	21 (47%)	0.2	>0.9
ARID1A	9 (64%)	18 (40%)	0.11	>0.9
KMT2D	9 (64%)	10 (22%)	0.007	>0.9
PIK3CA	9 (64%)	16 (36%)	0.057	>0.9
TCF7L2	8 (57%)	15 (33%)	0.11	>0.9
KRAS	6 (43%)	25 (56%)	0.4	>0.9
ASXL1	7 (50%)	9 (20%)	0.04	>0.9
FBXW7	7 (50%)	10 (22%)	0.087	>0.9
RNF43	7 (50%)	14 (31%)	0.2	>0.9
TP53	4 (29%)	22 (49%)	0.2	>0.9
B2M	6 (43%)	11 (24%)	0.2	>0.9
CREBBP	6 (43%)	5 (11%)	0.015	>0.9
CTCF	6 (43%)	5 (11%)	0.015	>0.9
RAD50	6 (43%)	12 (27%)	0.3	>0.9
MSH3	5 (36%)	17 (38%)	0.9	>0.9
MSH6	5 (36%)	17 (38%)	0.9	>0.9
ALK	5 (36%)	11 (24%)	0.5	>0.9
AMER1	5 (36%)	9 (20%)	0.3	>0.9
KMT2C	5 (36%)	8 (18%)	0.3	>0.9
BCORL1	4 (29%)	7 (16%)	0.4	>0.9
BRCA2	4 (29%)	6 (13%)	0.2	>0.9
CASP8	4 (29%)	5 (11%)	0.2	>0.9
CIC	4 (29%)	11 (24%)	0.7	>0.9
LRP1B	4 (29%)	8 (18%)	0.5	>0.9
MSH2	4 (29%)	2 (4.4%)	0.024	>0.9
NTRK3	4 (29%)	7 (16%)	0.4	>0.9
RET	4 (29%)	7 (16%)	0.4	>0.9
RPL22	4 (29%)	8 (18%)	0.5	>0.9
SMARCA4	4 (29%)	7 (16%)	0.4	>0.9
ARID1B	3 (21%)	6 (13%)	0.4	>0.9
ATR	3 (21%)	1 (2.2%)	0.038	>0.9
CDH1	3 (21%)	7 (16%)	0.7	>0.9
CTNNB1	3 (21%)	5 (11%)	0.4	>0.9
CYLD	3 (21%)	3 (6.7%)	0.14	>0.9
CYP1B1	3 (21%)	5 (11%)	0.4	>0.9
FLCN	3 (21%)	6 (13%)	0.4	>0.9
HDAC2	3 (21%)	2 (4.4%)	0.081	>0.9
NBN	3 (21%)	4 (8.9%)	0.3	>0.9

NOTCH1	3 (21%)	2 (4.4%)	0.081	>0.9
NRG1	3 (21%)	8 (18%)	0.7	>0.9
PBRM1	3 (21%)	5 (11%)	0.4	>0.9
PPM1D	3 (21%)	2 (4.4%)	0.081	>0.9
SETD2	3 (21%)	5 (11%)	0.4	>0.9
SMAD4	3 (21%)	3 (6.7%)	0.14	>0.9
ATM	2 (14%)	9 (20%)	>0.9	>0.9
PTEN	1 (7.1%)	9 (20%)	0.4	>0.9
BCL6	2 (14%)	8 (18%)	>0.9	>0.9
CHD2	2 (14%)	8 (18%)	>0.9	>0.9
HNF1A	2 (14%)	8 (18%)	>0.9	>0.9
UGT1A1	1 (7.1%)	8 (18%)	0.7	>0.9
EGFR	1 (7.1%)	7 (16%)	0.7	>0.9
ACVR1B	2 (14%)	4 (8.9%)	0.6	>0.9
APOB	2 (14%)	0 (0%)	0.053	>0.9
BARD1	2 (14%)	3 (6.7%)	0.6	>0.9
BCOR	2 (14%)	3 (6.7%)	0.6	>0.9
BRIP1	2 (14%)	0 (0%)	0.053	>0.9
CUX1	2 (14%)	4 (8.9%)	0.6	>0.9
EP300	2 (14%)	6 (13%)	>0.9	>0.9
FAS	2 (14%)	2 (4.4%)	0.2	>0.9
FAT1	2 (14%)	2 (4.4%)	0.2	>0.9
FBXO11	2 (14%)	3 (6.7%)	0.6	>0.9
FGF4	2 (14%)	1 (2.2%)	0.14	>0.9
FGFR3	2 (14%)	1 (2.2%)	0.14	>0.9
FLT4	2 (14%)	0 (0%)	0.053	>0.9
FUBP1	2 (14%)	2 (4.4%)	0.2	>0.9
GRIN2A	2 (14%)	2 (4.4%)	0.2	>0.9
ITPKB	2 (14%)	0 (0%)	0.053	>0.9
KDM6A	2 (14%)	0 (0%)	0.053	>0.9
MAP3K1	2 (14%)	3 (6.7%)	0.6	>0.9
MLH3	2 (14%)	3 (6.7%)	0.6	>0.9
NF1	2 (14%)	5 (11%)	0.7	>0.9
PALB2	2 (14%)	3 (6.7%)	0.6	>0.9
PAX5	2 (14%)	1 (2.2%)	0.14	>0.9
PMS2	2 (14%)	5 (11%)	0.7	>0.9
POT1	2 (14%)	0 (0%)	0.053	>0.9
PTCH1	2 (14%)	1 (2.2%)	0.14	>0.9
ZNRF3	2 (14%)	5 (11%)	0.7	>0.9
MLH1	1 (7.1%)	6 (13%)	>0.9	>0.9
ZFHX3	1 (7.1%)	6 (13%)	>0.9	>0.9
LATS1	1 (7.1%)	5 (11%)	>0.9	>0.9
RB1	0 (0%)	5 (11%)	0.3	>0.9

ARID2	0 (0%)	4 (8.9%)	0.6	>0.9
ERBB2	1 (7.1%)	4 (8.9%)	>0.9	>0.9
ERBB3	1 (7.1%)	4 (8.9%)	>0.9	>0.9
ERCC3	1 (7.1%)	4 (8.9%)	>0.9	>0.9
FGFR2	0 (0%)	4 (8.9%)	0.6	>0.9
MRE11	0 (0%)	4 (8.9%)	0.6	>0.9
MS4A1	0 (0%)	4 (8.9%)	0.6	>0.9
NCOR1	1 (7.1%)	4 (8.9%)	>0.9	>0.9
NKX2-1	0 (0%)	4 (8.9%)	0.6	>0.9
NTRK2	1 (7.1%)	4 (8.9%)	>0.9	>0.9
PTPN13	0 (0%)	4 (8.9%)	0.6	>0.9
RASA1	1 (7.1%)	4 (8.9%)	>0.9	>0.9
TSC1	0 (0%)	4 (8.9%)	0.6	>0.9
AKT1	1 (7.1%)	0 (0%)	0.2	>0.9
AR	1 (7.1%)	0 (0%)	0.2	>0.9
ATRX	1 (7.1%)	2 (4.4%)	0.6	>0.9
BAP1	1 (7.1%)	0 (0%)	0.2	>0.9
BCL10	1 (7.1%)	0 (0%)	0.2	>0.9
BCL11B	1 (7.1%)	2 (4.4%)	0.6	>0.9
BRCA1	1 (7.1%)	3 (6.7%)	>0.9	>0.9
CDC73	1 (7.1%)	2 (4.4%)	0.6	>0.9
CDK12	1 (7.1%)	2 (4.4%)	0.6	>0.9
CDKN1B	1 (7.1%)	3 (6.7%)	>0.9	>0.9
CDKN2A	1 (7.1%)	1 (2.2%)	0.4	>0.9
CHEK2	1 (7.1%)	2 (4.4%)	0.6	>0.9
CKS1B	1 (7.1%)	1 (2.2%)	0.4	>0.9
CPNE8	1 (7.1%)	0 (0%)	0.2	>0.9
CXCR4	1 (7.1%)	3 (6.7%)	>0.9	>0.9
DICER1	1 (7.1%)	2 (4.4%)	0.6	>0.9
DNM2	1 (7.1%)	0 (0%)	0.2	>0.9
DOCK8	1 (7.1%)	1 (2.2%)	0.4	>0.9
DPYD	1 (7.1%)	2 (4.4%)	0.6	>0.9
ERRFI1	1 (7.1%)	2 (4.4%)	0.6	>0.9
EZH2	1 (7.1%)	0 (0%)	0.2	>0.9
FANCB	1 (7.1%)	0 (0%)	0.2	>0.9
FGF10	1 (7.1%)	1 (2.2%)	0.4	>0.9
FGF3	1 (7.1%)	0 (0%)	0.2	>0.9
FGFR1	1 (7.1%)	0 (0%)	0.2	>0.9
GATA2	1 (7.1%)	0 (0%)	0.2	>0.9
GATA3	1 (7.1%)	0 (0%)	0.2	>0.9
GLI2	1 (7.1%)	3 (6.7%)	>0.9	>0.9
HCN1	1 (7.1%)	0 (0%)	0.2	>0.9
HNF1B	1 (7.1%)	2 (4.4%)	0.6	>0.9

HNMT	1 (7.1%)	3 (6.7%)	>0.9	>0.9
HSPH1	1 (7.1%)	0 (0%)	0.2	>0.9
ID4	1 (7.1%)	0 (0%)	0.2	>0.9
IDH2	1 (7.1%)	0 (0%)	0.2	>0.9
IFNAR1	1 (7.1%)	1 (2.2%)	0.4	>0.9
INPP4B	1 (7.1%)	1 (2.2%)	0.4	>0.9
INSIG2	1 (7.1%)	3 (6.7%)	>0.9	>0.9
JAK1	1 (7.1%)	3 (6.7%)	>0.9	>0.9
KEAP1	1 (7.1%)	0 (0%)	0.2	>0.9
KYAT3	1 (7.1%)	1 (2.2%)	0.4	>0.9
LOC285766	1 (7.1%)	0 (0%)	0.2	>0.9
MAP2K1	1 (7.1%)	2 (4.4%)	0.6	>0.9
MAP2K4	1 (7.1%)	3 (6.7%)	>0.9	>0.9
NRAS	1 (7.1%)	1 (2.2%)	0.4	>0.9
NSD1	1 (7.1%)	0 (0%)	0.2	>0.9
PAX8	1 (7.1%)	3 (6.7%)	>0.9	>0.9
PHLPP2	1 (7.1%)	1 (2.2%)	0.4	>0.9
PIK3R1	1 (7.1%)	3 (6.7%)	>0.9	>0.9
PLCG2	1 (7.1%)	0 (0%)	0.2	>0.9
PRDM1	1 (7.1%)	1 (2.2%)	0.4	>0.9
PTPRT	1 (7.1%)	2 (4.4%)	0.6	>0.9
RARA	1 (7.1%)	0 (0%)	0.2	>0.9
RICTOR	1 (7.1%)	0 (0%)	0.2	>0.9
RUNX1	1 (7.1%)	1 (2.2%)	0.4	>0.9
SDHA	1 (7.1%)	1 (2.2%)	0.4	>0.9
SDHB	1 (7.1%)	0 (0%)	0.2	>0.9
SLC35F5	1 (7.1%)	3 (6.7%)	>0.9	>0.9
SMAD2	1 (7.1%)	0 (0%)	0.2	>0.9
SMAD3	1 (7.1%)	3 (6.7%)	>0.9	>0.9
SMARCB1	1 (7.1%)	0 (0%)	0.2	>0.9
SMARCE1	1 (7.1%)	0 (0%)	0.2	>0.9
SOX9	1 (7.1%)	1 (2.2%)	0.4	>0.9
SPEN	1 (7.1%)	3 (6.7%)	>0.9	>0.9
STC1	1 (7.1%)	0 (0%)	0.2	>0.9
SUFU	1 (7.1%)	1 (2.2%)	0.4	>0.9
SUZ12	1 (7.1%)	2 (4.4%)	0.6	>0.9
SYK	1 (7.1%)	0 (0%)	0.2	>0.9
SYNE1	1 (7.1%)	2 (4.4%)	0.6	>0.9
TOP2A	1 (7.1%)	0 (0%)	0.2	>0.9
TTI2	1 (7.1%)	0 (0%)	0.2	>0.9
ZNF471	1 (7.1%)	0 (0%)	0.2	>0.9
ACVR1	0 (0%)	3 (6.7%)	>0.9	>0.9
APLNR	0 (0%)	3 (6.7%)	>0.9	>0.9

CDKN1A	0 (0%)	3 (6.7%)	>0.9	>0.9
DNMT3A	0 (0%)	3 (6.7%)	>0.9	>0.9
ERBB4	0 (0%)	3 (6.7%)	>0.9	>0.9
GNAS	0 (0%)	3 (6.7%)	>0.9	>0.9
MUTYH	0 (0%)	3 (6.7%)	>0.9	>0.9
MYL1	0 (0%)	3 (6.7%)	>0.9	>0.9
NOTCH3	0 (0%)	3 (6.7%)	>0.9	>0.9
TBX3	0 (0%)	3 (6.7%)	>0.9	>0.9
TET2	0 (0%)	3 (6.7%)	>0.9	>0.9
TNFAIP6	0 (0%)	3 (6.7%)	>0.9	>0.9
TSC2	0 (0%)	3 (6.7%)	>0.9	>0.9
ATIC	0 (0%)	2 (4.4%)	>0.9	>0.9
BAGE5	0 (0%)	2 (4.4%)	>0.9	>0.9
BRAF	0 (0%)	2 (4.4%)	>0.9	>0.9
CDKN2B	0 (0%)	2 (4.4%)	>0.9	>0.9
CTLA4	0 (0%)	2 (4.4%)	>0.9	>0.9
DAXX	0 (0%)	2 (4.4%)	>0.9	>0.9
DDX3X	0 (0%)	2 (4.4%)	>0.9	>0.9
DYNC2H1	0 (0%)	2 (4.4%)	>0.9	>0.9
ELF3	0 (0%)	2 (4.4%)	>0.9	>0.9
EPCAM	0 (0%)	2 (4.4%)	>0.9	>0.9
ESR1	0 (0%)	2 (4.4%)	>0.9	>0.9
FANCC	0 (0%)	2 (4.4%)	>0.9	>0.9
FGF23	0 (0%)	2 (4.4%)	>0.9	>0.9
FOXO3	0 (0%)	2 (4.4%)	>0.9	>0.9
IDH1	0 (0%)	2 (4.4%)	>0.9	>0.9
ING1	0 (0%)	2 (4.4%)	>0.9	>0.9
KDM5A	0 (0%)	2 (4.4%)	>0.9	>0.9
LDLR	0 (0%)	2 (4.4%)	>0.9	>0.9
LZTR1	0 (0%)	2 (4.4%)	>0.9	>0.9
MAGI2	0 (0%)	2 (4.4%)	>0.9	>0.9
MAX	0 (0%)	2 (4.4%)	>0.9	>0.9
MYB	0 (0%)	2 (4.4%)	>0.9	>0.9
NOTCH2	0 (0%)	2 (4.4%)	>0.9	>0.9
PHLPP1	0 (0%)	2 (4.4%)	>0.9	>0.9
SCART1	0 (0%)	2 (4.4%)	>0.9	>0.9
SH2B3	0 (0%)	2 (4.4%)	>0.9	>0.9
SLIT2	0 (0%)	2 (4.4%)	>0.9	>0.9
TBL1XR1	0 (0%)	2 (4.4%)	>0.9	>0.9
TP63	0 (0%)	2 (4.4%)	>0.9	>0.9
ABRAXAS1	0 (0%)	1 (2.2%)	>0.9	>0.9
ANO3	0 (0%)	1 (2.2%)	>0.9	>0.9
BCR	0 (0%)	1 (2.2%)	>0.9	>0.9

BIRC3	0 (0%)	1 (2.2%)	>0.9	>0.9
CACNA1B	0 (0%)	1 (2.2%)	>0.9	>0.9
CARM1	0 (0%)	1 (2.2%)	>0.9	>0.9
CCDC150	0 (0%)	1 (2.2%)	>0.9	>0.9
CCND2	0 (0%)	1 (2.2%)	>0.9	>0.9
CFTR	0 (0%)	1 (2.2%)	>0.9	>0.9
CHEK1	0 (0%)	1 (2.2%)	>0.9	>0.9
CUL3	0 (0%)	1 (2.2%)	>0.9	>0.9
DIS3L2	0 (0%)	1 (2.2%)	>0.9	>0.9
EBF1	0 (0%)	1 (2.2%)	>0.9	>0.9
FANCF	0 (0%)	1 (2.2%)	>0.9	>0.9
FANCI	0 (0%)	1 (2.2%)	>0.9	>0.9
FGF6	0 (0%)	1 (2.2%)	>0.9	>0.9
FHIT	0 (0%)	1 (2.2%)	>0.9	>0.9
FOXA1	0 (0%)	1 (2.2%)	>0.9	>0.9
FOXP1	0 (0%)	1 (2.2%)	>0.9	>0.9
GABRA6	0 (0%)	1 (2.2%)	>0.9	>0.9
GABRG2	0 (0%)	1 (2.2%)	>0.9	>0.9
GATA6	0 (0%)	1 (2.2%)	>0.9	>0.9
GLI1	0 (0%)	1 (2.2%)	>0.9	>0.9
GRM3	0 (0%)	1 (2.2%)	>0.9	>0.9
HGF	0 (0%)	1 (2.2%)	>0.9	>0.9
HSP90AA1	0 (0%)	1 (2.2%)	>0.9	>0.9
IGH	0 (0%)	1 (2.2%)	>0.9	>0.9
IKZF1	0 (0%)	1 (2.2%)	>0.9	>0.9
IQSEC3	0 (0%)	1 (2.2%)	>0.9	>0.9
IRF1	0 (0%)	1 (2.2%)	>0.9	>0.9
IRS1	0 (0%)	1 (2.2%)	>0.9	>0.9
ITM2C	0 (0%)	1 (2.2%)	>0.9	>0.9
JAK2	0 (0%)	1 (2.2%)	>0.9	>0.9
KLLN	0 (0%)	1 (2.2%)	>0.9	>0.9
LMNA	0 (0%)	1 (2.2%)	>0.9	>0.9
LMO1	0 (0%)	1 (2.2%)	>0.9	>0.9
MAP3K7	0 (0%)	1 (2.2%)	>0.9	>0.9
MAT2B	0 (0%)	1 (2.2%)	>0.9	>0.9
MEF2B	0 (0%)	1 (2.2%)	>0.9	>0.9
MEIS2	0 (0%)	1 (2.2%)	>0.9	>0.9
MEN1	0 (0%)	1 (2.2%)	>0.9	>0.9
MIB1	0 (0%)	1 (2.2%)	>0.9	>0.9
MITF	0 (0%)	1 (2.2%)	>0.9	>0.9
MTOR	0 (0%)	1 (2.2%)	>0.9	>0.9
MYH11	0 (0%)	1 (2.2%)	>0.9	>0.9
NCL	0 (0%)	1 (2.2%)	>0.9	>0.9

NF2	0 (0%)	1 (2.2%)	>0.9	>0.9
NOP10	0 (0%)	1 (2.2%)	>0.9	>0.9
NOTCH4	0 (0%)	1 (2.2%)	>0.9	>0.9
NSD2	0 (0%)	1 (2.2%)	>0.9	>0.9
PAX3	0 (0%)	1 (2.2%)	>0.9	>0.9
PDE4D	0 (0%)	1 (2.2%)	>0.9	>0.9
PDGFRA	0 (0%)	1 (2.2%)	>0.9	>0.9
PHF6	0 (0%)	1 (2.2%)	>0.9	>0.9
PIK3CB	0 (0%)	1 (2.2%)	>0.9	>0.9
PML	0 (0%)	1 (2.2%)	>0.9	>0.9
POLE	0 (0%)	1 (2.2%)	>0.9	>0.9
PPP2R1A	0 (0%)	1 (2.2%)	>0.9	>0.9
PPP2R2A	0 (0%)	1 (2.2%)	>0.9	>0.9
PRIM2	0 (0%)	1 (2.2%)	>0.9	>0.9
PRKAR1A	0 (0%)	1 (2.2%)	>0.9	>0.9
PSMD1	0 (0%)	1 (2.2%)	>0.9	>0.9
PTCH2	0 (0%)	1 (2.2%)	>0.9	>0.9
PTPN11	0 (0%)	1 (2.2%)	>0.9	>0.9
PTPRD	0 (0%)	1 (2.2%)	>0.9	>0.9
RAD51D	0 (0%)	1 (2.2%)	>0.9	>0.9
RAF1	0 (0%)	1 (2.2%)	>0.9	>0.9
RALGAPA2	0 (0%)	1 (2.2%)	>0.9	>0.9
RBM10	0 (0%)	1 (2.2%)	>0.9	>0.9
ROS1	0 (0%)	1 (2.2%)	>0.9	>0.9
SEC23B	0 (0%)	1 (2.2%)	>0.9	>0.9
SEC61G	0 (0%)	1 (2.2%)	>0.9	>0.9
SETBP1	0 (0%)	1 (2.2%)	>0.9	>0.9
SF3B1	0 (0%)	1 (2.2%)	>0.9	>0.9
SLITRK3	0 (0%)	1 (2.2%)	>0.9	>0.9
SMO	0 (0%)	1 (2.2%)	>0.9	>0.9
SPOP	0 (0%)	1 (2.2%)	>0.9	>0.9
STAT4	0 (0%)	1 (2.2%)	>0.9	>0.9
TCL1A	0 (0%)	1 (2.2%)	>0.9	>0.9
TENT5C	0 (0%)	1 (2.2%)	>0.9	>0.9
TGFBR1	0 (0%)	1 (2.2%)	>0.9	>0.9
TGFBR2	0 (0%)	1 (2.2%)	>0.9	>0.9
TMBIM1	0 (0%)	1 (2.2%)	>0.9	>0.9
TMEM60	0 (0%)	1 (2.2%)	>0.9	>0.9
TRAF7	0 (0%)	1 (2.2%)	>0.9	>0.9
TRIM51HP	0 (0%)	1 (2.2%)	>0.9	>0.9
WEE1	0 (0%)	1 (2.2%)	>0.9	>0.9
WNT10A	0 (0%)	1 (2.2%)	>0.9	>0.9
WNT5B	0 (0%)	1 (2.2%)	>0.9	>0.9

WNT6	0 (0%)	1 (2.2%)	>0.9	>0.9
WRN	0 (0%)	1 (2.2%)	>0.9	>0.9
ZMYM3	0 (0%)	1 (2.2%)	>0.9	>0.9

[a] Pearson's Chi-squared test

[b] False discovery rate correction for multiple testing

Supplemental S16: Tumor mutation burden among eoCRC (blue) and aoCRC (yellow) patients with an incidentally detected pathogenic/likely pathogenic germline variant in a Lynch syndrome gene.

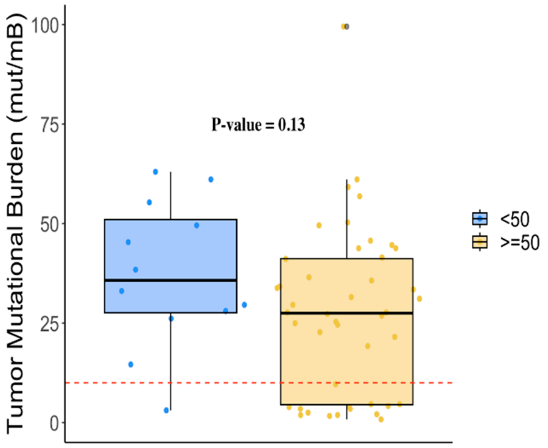

Supplement: Supplementary file 1 [file cancers-17-00836-s001.zip › cancers-3446894-supplementary.pdf]
